# Social Climate, Uncertainty and Fertility Intentions: from the Great Recession to the Covid-19 Crisis

**DOI:** 10.1007/s10680-023-09684-1

**Published:** 2023-12-01

**Authors:** Chiara Ludovica Comolli

**Affiliations:** Department of Statistical Sciences Paolo Fortunati, University of Bologna, Via Belle Arti 41, Bologna, 40126 Italy

**Keywords:** Fertility, Intentions, Crisis, Covid-19, Uncertainty, Social capital

## Abstract

The literature on fertility in context of crises considers major crises exclusively as economic experiences, however, they are also social phenomena, affecting communities, morality and social interactions. When changes in the social climate are of a sufficient magnitude, they tend to break down the social fabric and generate additional uncertainty, more of a social form, which may affect reproductive decisions beyond economic uncertainty alone. Applying Fixed Effects Models to 18 waves of the Swiss Household Panel (2004–2021), this study evaluates the relationship between changes in social climate and social uncertainty and first and second order childbearing intentions, net of economic uncertainty, sociodemographic determinants and unobserved time-invariant individual and local area characteristics. Canton-level mean and variance of generalized trust and optimism about the future are used as proxies of the quality and the unpredictability of the social climate respondents live in. Besides parity, the study explores period variation by comparing the time around the Great Recession (before, during and after) and the years around the Covid-19 pandemic. Results show that the worsening of the social climate and its growing uncertainty correlate with lower and more uncertain first and second birth intentions. Yet, important parity-period interactions emerge.

## Introduction

**Fig. 1:**
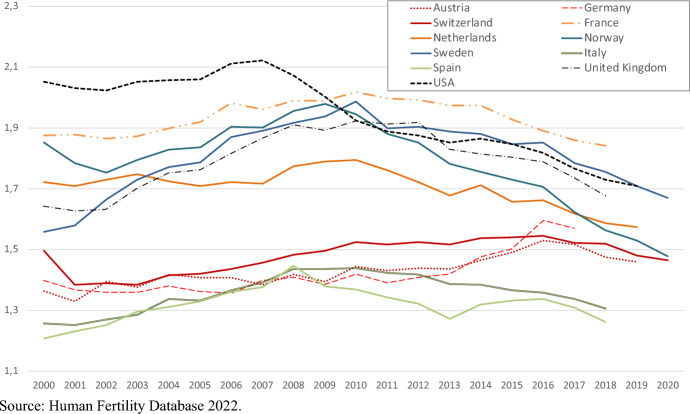
Total fertility rate in selected western countries (2000–2021). *Source* Human Fertility Database 2022

Persistently low fertility rates contribute to population aging, age structure imbalances and life course inequalities, posing challenges to the social and financial sustainability of contemporary western societies. Moreover, growing inequalities are driving a wedge between those who manage to reach their intended family size and those who do not (Mencarini et al., 2018). In most western countries, the 2008 economic crisis—the Great Recession—triggered a long period of declining birth rates (Sobotka et al., 2011a). Part of this fertility decline can be explained by the intensification of *economic uncertainty*, a condition in which future economic prospects cannot be deduced by present information (Dequech, 2000), that persisted long after the economic recovery. Economic uncertainty alone, however, does not explain why fertility in many countries (see Fig. 1) has declined for longer than a decade after the Great Recession, or why often the decline has even accelerated after 2015–2016, or, finally, why a strong fertility decline has characterized countries like the Nordic European which were only marginally affected by the crisis and the economic insecurity it generated (Comolli et al., 2021).

Against this background, in 2020 Covid-19 led to another crisis. The social, financial and labor market losses have been considerable, and the post-pandemic prospects remain highly uncertain. The Covid-19 crisis is first and foremost a health emergency, but individuals may also adjust their behavior to the considerable economic and social costs of the pandemic. As a result, fertility is likely to be affected too (Aassve et al., 2020, 2021a, 2021b; Charles-Edwards et al., 2021; Lappegård et al., 2020; Wilde et al., 2020). At least in the short-term, early evidence hints to a generally negative effect, although often short-lived and with contextual heterogeneities in direction and magnitude of the effect (Cohen, 2021; Emery & Koops, 2022; Nitsche et al., 2021; Sobotka et al., 2021). Furthermore, the possible mechanisms explaining the changes in reproductive decisions as a result of the pandemic are multiple but their unfolding is still unknown (Berrington et al., 2021; Castro Torres et al., 2022; Lindberg, 2021; Lindberg et al., 2020; Luppi et al., 2020).

The argument put forward in this study is that to understand the fertility trends witnessed in western countries in the past decade and to be able to anticipate if and how the pandemic will possibly influence such trends in the next decade, a paradigm shift is needed. While theories of the fertility response to business cycle fluctuations consider major crises exclusively as economic experiences, their effects are rarely confined to one domain. Economic downturns are also social phenomena. They have an impact on psychological outcomes, social interactions, communities and morality, thereby altering the social climate individuals live in. When these societal changes are of a sufficient magnitude, they tend to break down the social fabric and they represent additional sources of complexity generating a more *social* form of *uncertainty*, linked to changing expectations about the future of societies (Luhmann, 1979; Colquitt et al., 2012).

Despite possibly producing effects on reproductive decisions beyond those of economic uncertainty, the disruption of social practices has been largely overlooked by the literature on the determinants of low fertility in contemporary societies in the context of crises. Macro-level studies have previously uncovered the positive nexus between national or subnational levels of generalized trust and fertility rates, mostly seeing trust levels as invariable traits of societies that moderate the impact of time varying conditions or events on birth rates (Aassve et al., 2021a). Yet, others have shown that major events alter those traits. For instance, political sociologists have identified increasing economic and social inequalities and polarization, and declining access to different dimensions of social capital, as driving forces behind the increasing success of Radical Right Parties (RRP) in Europe (Rydgren, 2007). Comolli and Andersson (2021), in turn, have suggested that part of the lowered propensity of Swedish women to have a child during the last decade can be linked to the increasing support for the RRP (i.e., the Sweden Democrats) in the municipality in which they reside, and that the magnitude of the effect of the voting-support variable is similar to those observed for a more conventional indicator of economic uncertainty, i.e., unemployment rates. Recent studies in the field of health economics have demonstrated that the 1918 Spanish Flu permanently affected social trust levels (Aassve et al., 2021b) while, previously, other studies had showed that trust reacted to natural disasters, declining in case of earthquakes and floods (Albrecht, 2017; Calo-Blanco et al., 2017; Carlin et al., 2014; Uslaner & yamamura, 2016). Finally, social trust has been shown to decline in response to political crises, i.e., the collapse of communist societies (Rose-Ackerman, 2001). The current study fills a crucial gap in this literature by uncovering the response of individual-level reproductive decisions elicited by changes in the local social climate and in social, net of economic, uncertainties.

The study analyses social climate as the collective experience of a worsening of social spirit and practices in the local community, and social uncertainty as the increasing unpredictability of such social climate (using measures of distribution). Both are assumed to influence reproductive decisions insofar they negatively affect individuals’ view about the future of society and social relations. Yet, besides driving demographic processes, uncertainty represents a demographic outcome of inquiry. Non-numeric, or uncertain, answers have been previously shown to potentially represent rational responses to uncertain conditions (LeGrand et al., 2003; Trinitapoli & Yeatman, 2011; Hayford & Agadjanian, 2011). Uncertainty in fertility intentions is sizeable and should not be regarded as a residual category or measurement error but as an answer with substantive implications about reproductive uncertainty (Ni Bhrolchain & Beaujouan, 2011; Schaeffer & Thomson, 1992; Trinitapoli, 2023). Sharing this view, the current study investigates, besides positive numeric answers, also non-numeric (“Don’t Know”) responses to questions about short-term childbearing intentions^1^.

The study context is Switzerland and the data used come from the Swiss Household Panel (SHP). The SHP provides suitable data for this study for several reasons. First, the SHP stretches from 1999 to 2021, allowing to study longitudinal change over a long period of time including two major crises (the Great Recession and the Covid-19 Pandemic). The second advantage of the SHP data is that it collects repeated information about short-term fertility intentions in each wave starting from 2002 and the number of children ever had from a representative sample of household from all cantons in Switzerland. This allows investigating parity-specific intentions, important insofar childbearing decisions are made sequentially and the determinants of reproductive decisions differ between the first and higher-order births (Kreyenfeld, 2021). Third, the SHP reports respondents’ canton of residence, making it possible to measure the social climate and its unpredictability in the local area surrounding the respondents. Finally, the SHP collects information on economic conditions and perceptions and on generalized trust yearly from 2004.

Local levels and variance of generalized trust in the canton of residence are used here as one set of proxies of perceived social climate and uncertainty. Ideally, being uncertainty essentially about the future, one would like to operationalize social uncertainty through information about the *expectations about the future of social relations, reciprocity and morality*. Unfortunately, to the best of the author’s knowledge no existing survey asks such questions. However, the European Social Survey (ESS) collects information about pessimism/optimism about the future of the world through the agreement/disagreement to the question: “The way things are now, I find it hard to be hopeful about the future of the world.” The measure does not specifically refer to the social realm and it was unfortunately collected only in two cross-sectional waves (2006 and 2012). However, “Optimism about the future”—merged with the SHP data at the wave-by-canton level—will serve as a benchmark for our indicator directly linked to the social climate (i.e., generalized trust) as it makes explicit reference to future expectations, a key component of the operationalization of uncertainty. Local levels and variance of Optimism about the future thus represent the second set of proxies of perceived social climate and uncertainty.

First, using (individual and canton) Fixed Effects models, I analyze the association between changes over time in local levels and distribution of generalized trust and optimism about the future and the probability of expressing positive or uncertain intention to have a(nother) child among Swiss partnered childless and one-child parents. Fixed Effects models allow to additionally control for unobserved time-invariant individual and canton characteristics that influence both social climate and uncertainty and the intention to have a first or a second child. Second, disposing of almost two decades of data, I will be able to investigate whether these associations vary across periods and in particular across the two major crises episodes that characterized the recent history of western countries: The Great Recession and the Covid-19 pandemic.

## Theoretical Background and Empirical Evidence

### Economic Conditions, Economic Uncertainty and Fertility

One of the strongest driving forces of fertility is represented by individuals’ economic prospects. The New Home Economics (NHE) theory sees childbearing as a rational choice based on the costs and benefits of children and posits that a decline in households’ income is linked to the postponement of childbearing (Becker, 1993). When incomes drop and unemployment rates rise, long-term commitments—such as housing purchases or having children—tend to be postponed. The impact of employment status on the entry into parenthood has been widely investigated in the past, and increasingly so after the onset of the Great Recession. Theoretical arguments and empirical evidence show that economic and labor market instability are important factors explaining the postponement of family formation in contemporary society (Kreyenfeld & Andersson, 2014; Kreyenfeld et al., 2012). Some studies focusing on the Great Recession demonstrate that not only behavior but also fertility intentions are negatively affected economic downturns (Testa & Basten, 2014; Fiori et al., 2018; Novelli et al. 2021).

Yet, objective economic conditions are not the unique rationale of childbearing: preferences, social norms and values (Lesthaeghe & Van de Kaa, 1986) and institutional and structural constraints (Esping-Andersen & Billari, 2015) influence childbearing decisions. Furthermore, the notion of *economic uncertainty* has become central to the sociodemographic literature on fertility in the aftermath of the Great Recession. Perceived economic uncertainty, beyond material economic conditions, produces a negative re-evaluation of current and future economic prospects that operates as an additional driver of the postponement of irreversible commitments, such as having children (Comolli & Vignoli, 2021; Ranjan, 1999; Vignoli et al., 2020a, 2020b). The recent application of the Narrative Framework to fertility decisions emphasizes the role of objective conditions as the “shadow of the past,” and subjective conditions as “imagined futures” or “shadows of the future” (Bernardi et al., 2019, p. 4; Vignoli et al., 2020a, p. 26). Despite the ameliorating objective economic conditions in the second half of the 2010s, persisting perceived uncertainties seem to have reinforced the pessimistic evaluation of future economic prospects, which may have induced a continued postponement of irreversible transitions, such as having a child (Kreyenfeld et al., 2012; Schneider, 2015; Hofmann et al., 2017). A number of studies demonstrates that, ahead of behavior, perceived economic uncertainty influences fertility intentions (Busetta et al., 2019; Fahlén & Olàh, 2018; Modena & Sabatini, 2012).

Despite this conceptual advancement, it remains unclear why fertility declines were so widespread in western countries, still so even more than ten years after the Great Recession and at an accelerated decline in the second half of the 2010s, including in regions (e.g., Central and Northern Europe) only marginally affected by the Great Recession. Despite the rich amount of research, our knowledge about the determinants of the recent prolonged fertility decline remains incomplete, perhaps because studies have focused disproportionately on the purely economic consequences of the Great Recession and crises in general.

### Social Climate, Social Uncertainty and Fertility

Individuals are embedded in a social context (Elder, 1974; Entwisle, 2007) and use community actions as sources of information to navigate complex situations (Montgomery & Casterline, 1996; Rossier & Bernardi, 2009). Yet, social interactions do not only produce information but also produce resources or social capital^2^ (Coleman, 1990). Recently, social psychologists and political scientists have shown that social capital tends to be affected by long-lasting periods of lower opportunities (Ayllon, 2019; Matsudaira, 2016). Enduring inequalities lower civic spirit, trust, and civic engagement, and correlate with political polarization (Giustozzi & Gangl, 2021; Shayo, 2009; Uslaner & Brown, 2005). Economists and sociologists have come to similar conclusions: inequality causes marginalization and reduces social trust (Kearney & Levine, 2014). This may happen because the greater the distance between individuals at the top and the bottom, the less the different strata share a common fate and trust each other, and because “trust rests on a psychological foundation of optimism and control over one’s own environment” (Uslaner & Brown, 2005, p. 869), which inequality reduces. Yet, inequality is only one of the mechanisms through which major crises may affect morality and trust. Institutional failures in managing crises represent an alternative mechanism witnessed in the case of historical epidemics and natural disasters (Aassve et al., 2021b; Carlin et al., 2014) which may also apply to economic crises. The prioritization of individuals’ personal interest over the achievement of collective goals and community protection may represent another explanation of why trust and social integration decline among individual affected by major disruptions (Albrecht, 2017).

Social capital, in turn, is used by individuals as a crucial strategy to reduce the complexity arising from the burden of the multiplicity of possible events in society (Luhmann, 1979), and to mitigate existing uncertainty (Colquitt et al., 2012). Participating in community life and social activities provides information that diminish uncertainty. A high level of generalized trust and, more generally, a more positive social climate instills a sense of comfort that mitigates uncertainty (Arpino & Obydenkova, 2020), while the shredding of good social practices and generalized morality induces a sense of insecurity regarding the future of societies and social interactions. Individuals face a fundamental dilemma: while both individuals’ and societies’ outcomes are maximized by cooperation and social participation, it also makes individuals vulnerable to exploitation as some cooperative actions might not be rewarded (Kollock, 1998). A more hostile social climate contributes to greater social complexity and to generate a sense of social uncertainty that pervades life, particularly during times of societal change (Lind, 2001).

In a very recent book, offering a comprehensive theoretical framework to reconcile the literature on *uncertainty demography*, Jenny Trinitapoli presents a working definition of uncertainty, that covers all the different dimensions of uncertainty as experienced by the individual: “Uncertainty refers to epistemic situations in which the salience of the unknown and the unknowable eclipses the relevance of known factors” (Trinitapoli, 2023, p. 76). Uncertainty can be both individually perceived (Mills & Blossfeld 2003; Kreyenfeld et al., 2012; Alderotti et al. 2021) and a collective factor, reflecting a more unconscious phenomenon that goes beyond person-specific circumstances and relates to the overall aggregate climate (Comolli & Vignoli, 2021; Sobotka et al., 2011a, 2011b; Vignoli et al., 2020b). Individuals’ decisions and actions, through structures of social interaction, are informed by a constant macro–micro feedback (Elder, 1974; Entwisle, 2007; Huinink & Kohli, 2014). As contextual conditions vary, the perception of the individual situation or the response to such situation may change accordingly, for example, because contextual circumstances provide information about other individuals’ situation or about one’s own possible future situation (Comolli, 2021; Kreyenfeld, 2010). What is crucial is that uncertainty is measurable and has to be measured, if not directly, through proxies (Trinitapoli, 2023).

**Fig. 2:**
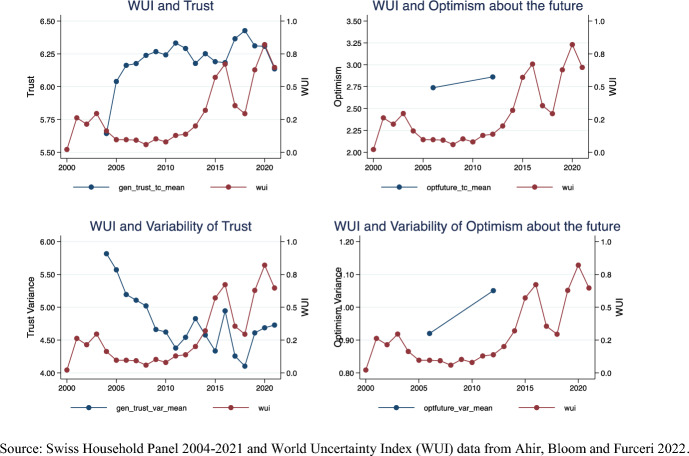
World Uncertainty Index (WUI) and optimism and generalized trust (national mean and variance) in Switzerland (2000–2021). *Source* Swiss household panel 2004–2021 and World Uncertainty Index (WUI) data from Ahir et al., 2022

Here I proxy social climate with local-level *mean* of generalized trust and optimism about the future and social uncertainty with the predictability of such mean levels, using local-level *variance* of trust and optimism. These proxies correlate with macro-level indexes of uncertainty. Figure 2 plots the World Uncertainty Index (WUI)^3^ in Switzerland in 2000–2021 (Ahir, Bloom & Furceri, 2022), together with aggregated local mean and variance of generalized trust and optimism about the future (SHP and ESS data). As in other western countries, where uncertainty is strongly synchronized (Ahir et al., 2022), the WUI in Switzerland (red line) peaks around major events like the economic crisis of the early 2000s (together with the idiosyncratic shock of a referendum in Switzerland deciding in favor of joining the UN in late 2002), the Euro Sovereign Debt crisis in 2011–13 (more than during the early phase of the financial crisis in 2009), the Brexit vote in 2016 (together with the idiosyncratic shock in Switzerland of a possible referendum to limit the number of EU immigrants) and the 2020 Covid-19 pandemic. Those peaks in uncertainty are usually accompanied by drops in levels and rises in the variability of trust and optimism about the future. This is clearly no proof of a causal relationship between them, but it suggests that the social climate tends to worsen with rising uncertainty while the unpredictability of the social climate tends to increase with rising uncertainty.

Social capital is a powerful determinant of fertility (Balbo et al., 2013). The positive link between generalized trust and fertility has been previously investigated in macro-level studies (Aassve et al., 2018, 2021a, 2021b). Where morality is more limited, fertility rates are lower because of lower quality of institutions (i.e., childcare provisions), the lower likelihood of couples to marry and to outsource childcare (Aassve et al., 2016; Cherlin et al., 2016). One defining feature of these studies though is that different dimensions of social capital and social trust have mainly been interpreted as time-invariant fixed traits of groups or societies that are transmitted “from generation to generation” (Aassve et al., 2018, pp. 4–5). In contrast, the abovementioned literature highlights that major crises may alter those traits. Societal changes that work to break down the social fabric and lower the levels of generalized trust represent additional sources of uncertainties, related to the future of individuals’ social support and societal values as well as the perceived future quality of social institutions. A recent study suggests that part of the lowered propensity of Swedish women to have kids during the last decade can be linked to the increasing support for the RRP of the Sweden Democrats, which the authors use as a proxy of the increasing social uncertainty in women’s area of residence (Comolli & Andersson, 2021). With the exception of Comolli and Andersson (2021), the hypothesis that crises generate not only economic but also social uncertainties and that the latter too drive contemporary childbearing decisions has never been explored.

## The Current Study

### Context

**Fig. 3:**
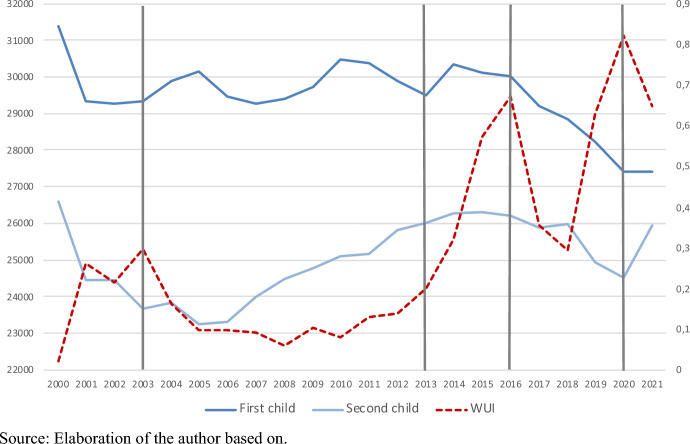
Live births by parity and World Uncertainty Index (WUI) in Switzerland, 2000–2021. *Source* Elaboration of the author based on

The study context is Switzerland, a country characterized by persistently low fertility rates (Total Fertility Rate at 1.46 in 2020, see Fig. 1) and high rates of childlessness (above 20% in the birth cohorts 1950s-1960, Sobotka, 2017). Figure 3 plots live births by parity (first and second) in 2000–2021 and the WUI. As in other contexts, the recent fertility declines are concentrated on first births and after 2014 (coinciding with the first strong peak in WUI). After years of increase, second births also decline after 2018 (around the second peak in WUI).

The Swiss value stability as a condition for life and family planning (Hanappi & Lipps, 2019) but labor protection of both permanent and temporary workers is weaker in Switzerland than in other German-speaking countries (OECD, 2016). Moreover, while men work almost universally in full-time jobs, women work mostly part-time (Levy et al., 2006; Sobotka et al., 2011b). Switzerland’s incentives for a traditional male breadwinner–female caretaker division further include generous dependent tax allowances, household instead of individual taxation, and high marginal tax rates that penalize second earners (Cooke & Baxter, 2010). In addition, in Switzerland work–family reconciliation policies are poor: childcare is extremely expensive and its provision insufficient to meet the demand which often leads to mothers leaving the labor market to take care of their children (Gauthier & Philipov, 2008; Wall & Escobedo, 2013)^4^. Overall, Switzerland displays great gender divides in family responsibilities that relapse almost entirely on women, who end up with a weaker and irregular labor market attachment over the life course^5^. The birth of a child is hence a career disruption that many women consider carefully. In such a context, perceived economic uncertainty may be due to worries of losing a job even if on a permanent contract, or due to (especially women’s) worries of not being able to balance work with childrearing and care duties.

In international comparison, Switzerland fared relatively better than its bordering countries during the Great Recession. Gross Domestic Product (GDP) growth sank at -2% in 2009 but quickly recovered the year after, although suffering in 2011–12, during the Sovereign Debt crisis in Europe and never returning to the growth levels of the pre-crisis years (2004–08). Unemployment in Switzerland tends to be low but, after having reached 4.8% in 2010, it stabilized around that share, peaking again at 4.92% in 2016. During the pandemic, the situation looked worse: a record negative GDP growth was registered (−2.4%) and in April 2020 unemployment increased by around 43% relative to the previous year, rising “almost as much as it increased in all of 2010 following the financial crisis” (Sheldon, 2020, p. 1). The prime difference between the two periods concerns the number of employees put on short-time work (furlough pay): about 1.9 million Swiss workers (more than one out of three) by the end of April 2020, compared to the below 100,000 workers put on short-time in June-July 2009 (Faber et al., 2020; Kopp & Siegenthaler, 2021).

**Fig. 4:**
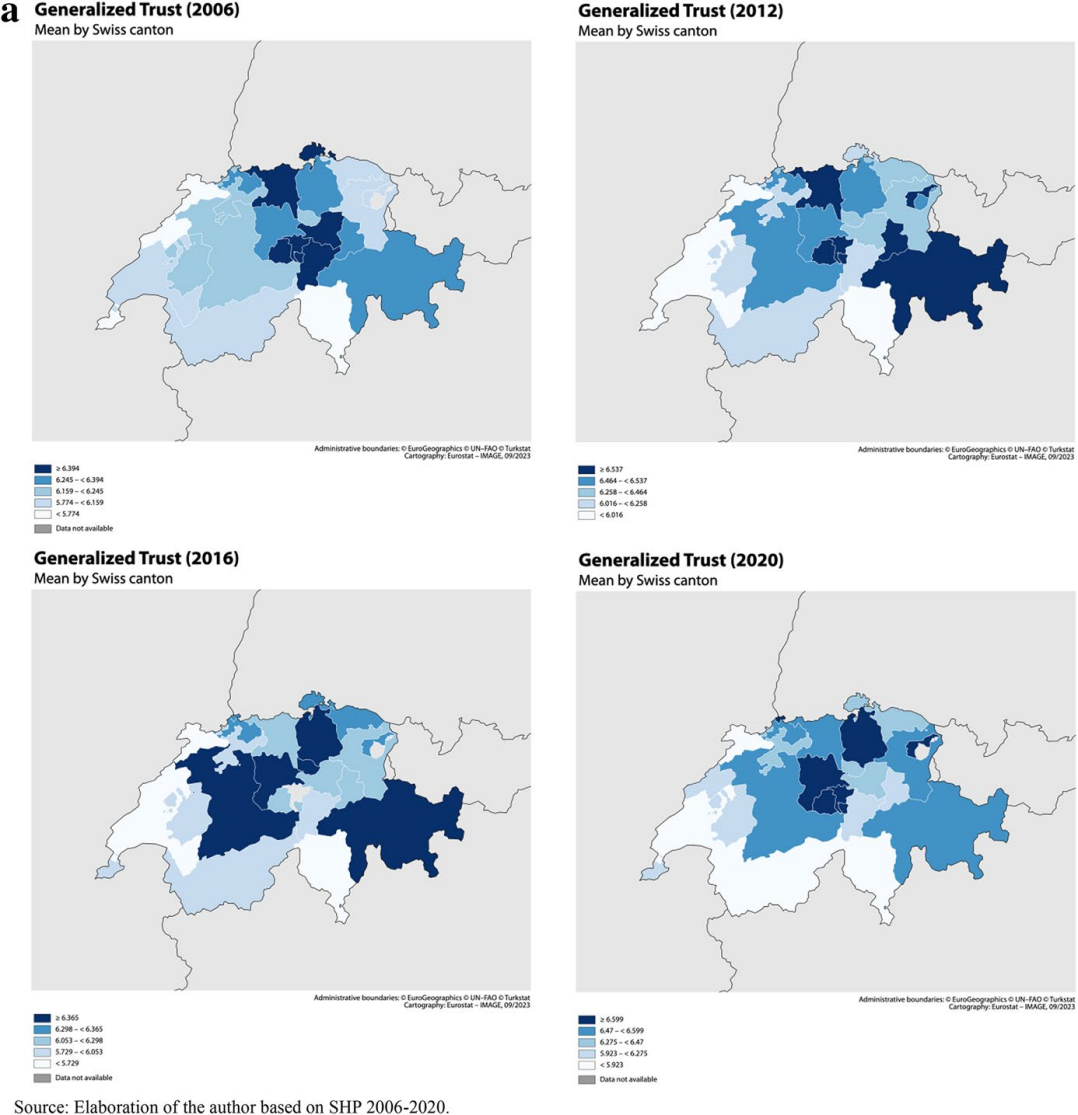

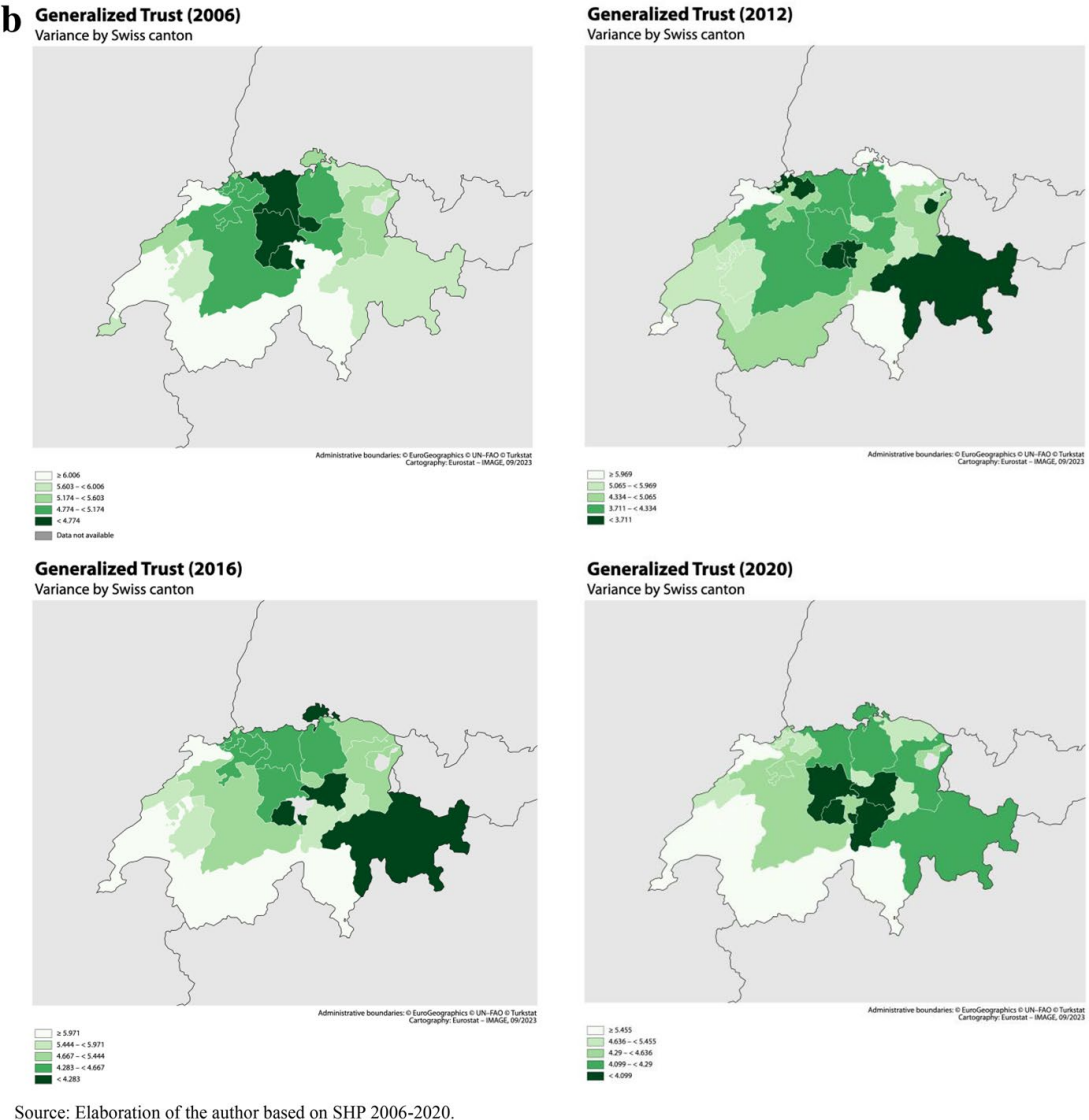

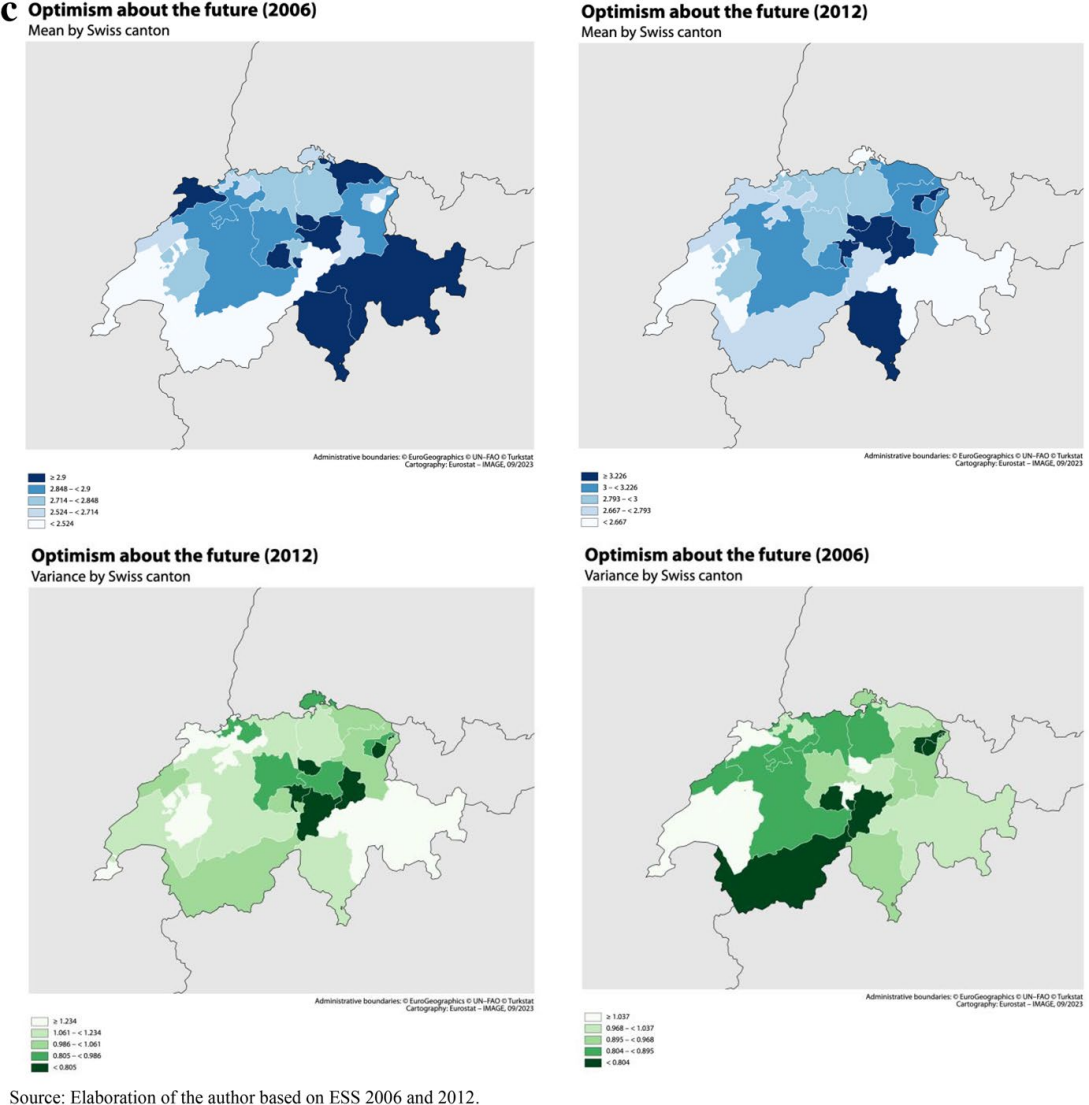
**a** Map of Mean Generalized trust by Swiss canton. Selected years. *Source* Elaboration of the author based on SHP 2006–2021. **b** Map of Variance of Generalized trust by Swiss canton. Selected years. *Source* Elaboration of the author based on SHP 2006–2021. **c** Map of Mean and Variance of Optimism about the future by Swiss canton (2006 and 2012). *Source* Elaboration of the author based on ESS 2006 and 2012

Finally, Switzerland ranks comparatively high in terms of social capital, being closer to Northern European countries in that respect than to continental Europe (Freitag, 2003; Wollebæk & Strømsnes, 2008). However, regional differences are important within the country. German-speaking regions tends to display higher levels of social capital than French-speaking, both followed by Italian-speaking regions (Freitag, 2003). The determinants of the relatively high levels of trust are more related to attitudes, resources and socioeconomic characteristics than to high levels of civic engagement. The Swiss institutional context also plays an important role: the political system and culture, characterized by direct democracy and a strong subsidiary principle, favors trust towards both political actors and fellow citizens (Freitag, 2003). Figure 4a, 4b and 4c presents maps of the social climate and social uncertainty measures by canton in selected years (SHP data). Besides the overall linguistic regional differences depicted above we observe both a large heterogeneity by canton within regions and a large fluctuation over time in both canton-level mean and variance of generalized trust and optimism. This geographical and time heterogeneity is exploited in this study to answer the research questions presented below.

### Research Questions

*The first research question asks whether changes in social climate and social uncertainty influence childbearing intentions beyond their known determinants.* The hypothesis is that, net of sociodemographic and economic, objective and subjective, factors, both a worsening of the social climate and its growing unpredictability induce a postponement of the decision to have a (nother) child. Such postponement may materialize either as a reduction in the (certain) probability of wanting a child in the near future, or as greater uncertainty about wanting a child in the near future (Ni Bhrolchain & Beaujouan, 2011; Schaeffer & Thomson, 1992; Trinitapoli, 2023). Yet, important heterogeneities across parity transitions exist. Compared to a second (or third) child, the transition to parenthood is particularly time-intensive and financially demanding, and it entails a life-changing decision (Kreyenfeld, 2021). More than higher parities, in fact, first births have been postponed as a result of growing economic uncertainty during and after the Great Recession period (Sobotka et al., 2011a). In contrast, the sudden and prolonged cut to the outsourcing of family care services during the pandemic has affected parents of small kids disproportionally (Collins et al., 2021; Cook & Grimshaw, 2021; Hipp & Bünning, 2021; Steiber et al., 2021). Pooling all parities together may thus mask such differences so *I analyze the association between social climate and social uncertainty and the intention to have a child separately for the first and the second*.

*The second research question asks whether the relationship between changing social climate and social uncertainty and childbearing intentions differs across periods.* In Switzerland as in other western countries, the study period, in fact, is characterized not only by a varying trend in first and second births intensities, but also by substantial ups and downs in indexes of national uncertainty (WUI) and by the unfolding of two major international crises: the Great Recession and the Covid-19 pandemic. While Covid-19 has possibly led to even larger economic losses and greater economic uncertainty than the Great Recession did, especially in Switzerland (Baker et al., 2020; Faber et al., 2020), the two crises differ in a number of ways. On the one hand, because of the confinement measures adopted to contain the spread of the virus, the alteration of the social climate and the growing uncertainty have been more immediately evident during the Pandemic than during the Great Recession, which may lead to observe in 2020 even stronger effects on childbearing decisions. On the other hand, the nature of the pandemic is more exogenous, which may have led individuals, first, to think that the crisis would be followed by a quicker recovery than those observed during the 2010s decade and, second, to diminish the perception of an institutional failure in managing the crisis, compared to the Great Recession. These pandemic features may lead thus to weaker effects of the pandemic on childbearing decisions. Finally, because of the confinement measures, parents have been disproportionately affected compared to the childless during the Pandemic relative to the Great Recession, therefore different parity dynamics are likely to emerge during the two crises.

### Data

To answer the research questions presented above I use the 2004–2021waves of the Swiss Household Panel (SHP) (ids 28,498, *N* = 161,807). The SHP is a nationally representative longitudinal survey of private households in which all adult (age 14 +) members are interviewed annually. To moderate attrition, three refreshment samples, in 2004, 2013 and 2020, were added to the initial sample of 1999. As a result, both attrition and non-response bias remain rather low concerning demographic and socioeconomic variables (Voorpostel & Lipps, 2011). These data are useful for the current study for a number of reasons. First, longitudinal data are needed to investigate how changes over time in the explanatory variables of interest affect childbearing decisions, and the SHP provide such data. Second, the SHP covers a very long period of time including the pre and post Great Recession years up to the most recent data collected during the Covid-19 pandemics in 2020 and 2021^6^. Third, the SHP combines household data with rich yearly individual information on demographic events, fertility intentions, objective employment conditions and subjective perceptions of job insecurity, plus the variable on which the first proxy of social climate and social uncertainty is based, generalized trust. Finally, the SHP provides information regarding respondents’ canton of residence (see Table 2 for distribution of observations by canton). This allows the identification of local areas of residence and the operationalization of the social climate respondents are exposed to. Through the respondents’ canton of residence, I also merge the second variable used to proxy social climate and social uncertainty, namely Optimism about the future, collected in the 2006 and 2012 European Social Survey (ESS) which also reports respondents’ canton of residence.

### Analytic Sample

The starting analytical sample consists of respondents in reproductive age 15–44 interviewed between 2004 and 2021 (14,666 ids, *N* = 63,914). First, among those, given the relatively short-time horizon presented for the childbearing intention questions (24 months), only partnered individuals are selected (11,070 ids, *N* = 43,221), the majority of them co-residing with their partners (74.3%). Second, missing or inapplicable observations (*N* = 2097), and observations from parents with already two or more children (*N* = 16,966) or with adult children are dropped (*N* = 1097). Third, observations from respondents with children other than their own in the household are removed (*N* = 1977 among childless and *N* = 175 among one-child parents). Finally, once observations from individuals who do not want children at all 7 are excluded (*N* = 3143), I obtain a sample of 5663 individuals (2610 men and 3,053 women; *N* = 17,766) observed between 1 and 18 times (the average number of waves of participation is 9.6) who are either childless (4547 ids, *N* = 13,272, 74.7% of total observations) or parents of one child (1752 ids, *N* = 4494, 25.3%). The sample for the analyses on Optimism about the future is substantially smaller since only the 2006 and 2012 waves can be used (1355 ids, *N* = 1537; 1024 childless respondents, *N* = 1149 and 349 parents, *N* = 388).

### Variables

The outcome of inquiry is whether a *child is wanted in the next 24 months* conditional on ever wanting any children. The original variable is categorical with three possible answers: *Yes*, *No*, or *Don’t know*. From that I derived a first dependent variable: the probability of holding positive childbearing intentions conditional on giving a numeric (Yes/No) answer, and a second dependent variable: the probability of non-numeric (Don’t Know) over numeric (Yes/No) answers.

The key explanatory variables are social climate and social uncertainty. The experiences of social climate and social uncertainty are operationalized, respectively, through the aggregate local (in the canton of residence for each wave in the analysis) level (mean) and variability (variance) of the individual-level variable^8^. In the SHP, the respondent’s generalized trust is measured through the question of whether *Most people can be trusted or you can*’*t be too careful in dealing with people* (original answers in the scale 0–10: from Can’t be too careful to Most people can be trusted). The variable Optimism about the future is constructed based on the question: *The way things are now, I find it hard to be hopeful about the future of the world* (original answers on a scale 0–10 from completely agree to completely disagree so the greater the answer the more optimistic is the respondent). As with trust, individual responses are aggregated by canton-wave and the so constructed means and variance are merged by canton-wave to the SHP data.

The other independent variable is perceived individual-level economic uncertainty measured through “cognitive” self-reported job insecurity, which denotes an employed person’s assessment of how secure (very secure, quite secure, a bit insecure, very insecure) his or her job is (Anderson & Pontusson, 2007; Esser & Olsen, 2012). Due to the low frequency of extreme answers, the variable has been recoded as binary: having a “very or quite secure” job versus having a job that is “a bit or very insecure”. The role of economic uncertainty is evaluated net of objective economic conditions measured through men’s and women’s employment status. Employment status includes the categories of employed, unemployed, out of the labor force^9^ and students. Because the job insecurity question is asked only to employed respondents, the employment status and job insecurity variables are recoded together as Employment Uncertainty, comprising the categories of: Employed with secure job, Employed with insecure job, Unemployed, Out of the labor force, or Student.

Finally, a number of control variables are included: respondents’ age (categorical, by 5 years age-groups), educational level (categorical: primary or lower secondary; upper secondary or tertiary education), disposable income (in thousand Swiss Francs, mean-centered) and dummies for cantons to control for other time-invariant characteristics of the local area respondents live in that possibly influence both social climate and fertility intentions. A survey period variable is included first to control for possible time trends and later to investigate possible period interactions (categorical: years before the GR 2004–2008; GR years 2009–2013; post GR years 2014–2018; years around the pandemic 2019–2021^10^. In models for parents, the age of the first child (mean-centered) is additionally controlled for.

### Method

To exploit the longitudinal character of the data and properly investigate change over time, I run within individual-within canton Fixed Effects (FE) Linear Probability models. FE models control for unobserved (time invariant) individual and canton characteristics influencing both the outcome, childbearing intentions, and the explanatory variables, namely local social climate and uncertainty and individual-level economic uncertainty. I analyze separately the change over time in the probability of wanting a child, conditional on giving a numeric/certain answer (Yes/No), and the probability of giving a non-numeric/uncertain answer (Don’t know).

In a first set of the analyses, I run—separately by indicator and parity (childless and one-child parents)—several step-wise models of the change over time in the probability of expressing a given intention to have a (nother) child during the following two years. Model 1 only controls for respondents’ age, Model 2 controls for all sociodemographic determinants and Model 3 adds employment uncertainty. In a second set of analyses I investigate the variation by period. To ease the interpretation, results are presented graphically through average marginal effects and predicted probabilities of positive and uncertain answers. Complete tables are provided in the Appendix.

## Results

Table 1 reports variables’ descriptive statistics by parity (childless and parents of one child) and models (Trust models with full 2004–2021 waves and Optimism models restricted only to the 2006 and 2012 waves). The vast majority of childless respondents (around 70% in both models) do not intend to have a first child in the following two years, while 50–60% of respondents do not intend to have a second child in the following two years. One fourth of respondents hold on average positive intentions about the first child while around 40% intend to have a second child in the next two years. Uncertain answers vary from around 2.9% among the childless in the optimism models to 4.9% among the parents of one child in the trust models. In general, parents tend to have more positive and more certain short-term childbearing intentions compared to childless. Parents are also older on average (around 36 years old versus 28 of the childless) and they tend to have higher education, to be more often employed and with more secure jobs compared to childless, with the exception of being out of the labor force. The 8–9% share of non-working parents is an average between genders. Mothers are around ten times more likely to be inactive compared to fathers (12.5–16% vs. 1.5–1.8% depending on models, not shown). No gender difference exists instead among inactive childless respondents.

**Table 1:** Descriptive statistics *Source* Elaboration of the author based on SHP 2004–2021

(a)								
	Trust models (waves 2004–2021)				Optimism models (waves 2006, 2012)			
	Childless		Parents		Childless		Parents	
	*N*	%	*N*	%	*N*	%	*N*	%
Child wanted in the next 24 months								
No	9288	69.98	2336	51.98	826	71.89	229	59.02
Yes	3343	25.19	1938	43.12	290	25.24	143	36.86
Does not know (D/K)	641	4.83	220	4.90	33	2.87	16	4.12
Total	13,272	100.00	4494	100.00	1149	100.00	388	100.00
Employment status + Job insecurity								
Employed, Very or quite secure	10,436	78.63	3598	80.06	886	77.11	302	77.84
Employed, A bit or very insecure	1229	9.26	438	9.75	103	8.96	38	9.79
Unemployed	363	2.74	88	1.96	36	3.13	8	2.06
Out of Labor Force	286	2.15	351	7.81	25	2.18	38	9.79
Students	958	7.22	19	0.42	99	8.62	2	0.52
Total	13,272	100.00	4494	100.00	1149	100.00	388	100.00
Age								
15–19	750	5.65	8	0.18	91	7.92	1	0.26
20–24	3670	27.65	81	1.80	338	29.42	11	2.84
25–29	4178	31.48	393	8.74	333	28.98	20	5.15
30–34	2714	20.45	1241	27.61	202	17.58	98	25.26
35–39	1243	9.37	1394	31.02	105	9.14	117	30.15
40–44	717	5.40	1377	30.64	80	6.96	141	36.34
Total	13,272	100.00	4494	100.00	1149	100.00	388	100.00
Educational level								
Primary or Low Sec	1300	9.80	222	4.94	148	12.88	18	4.64
Upper Secondary	6271	47.25	2038	45.35	564	49.09	188	48.45
Tertiary	5701	42.96	2234	49.71	437	38.03	182	46.91
Total	13,272	100.00	4494	100.00	1149	100.00	388	100.00
Wave								
2006					457	39.77	163	42.01
2012					692	60.23	225	57.99
Total					1149	100.00	388	100.00

(b)										
Trust models										
	Childless					Parents				
	*N*	Mean	SD	Min	Max	*N*	Mean	SD	Min	Max
Local area generalized trust mean	13,272	6.23	.56	0	10	4494	6.20	.54	2	8.75
Local area generalized trust variance	13,255	4.71	1.62	0	40.5	4491	4.71	1.56	0	16.33
Income°	13,272	9.24	5.82	0	191.94	4494	9.19	5.27	0	180.54
Age of first child	13,272	.003	.21	0	17	4494	4.29	4.36	0	17
Wave	13,272	2013.86	5.09	2004	2021	4494	2013.8	5.06	2004	2021

(c)										
Optimism models										
	Childless					Parents				
	*N*	Mean	SD	Min	Max	*N*	Mean	SD	Min	Max
Local area optimism about the future mean	1149	2.81	.24	2	3.48	388	2.81	.26	2.28	3.48
Local area optimism about the future variance	1149	.99	.15	0	1.38	388	1.01	.15	.5	1.88
Income°	1149	9.08	5.15	0	40.31	388	9.48	4.46	0	30.45
Age of first child	1149	0	0	0	0	388	4.94	4.52	0	17

The table reports summary statistics based on the sample that includes all childbearing intentions. Models for the probability of positive intentions are ran conditional on certain answers, therefore, on a restricted sample that excludes the D/K observations

**Fig. 5:**
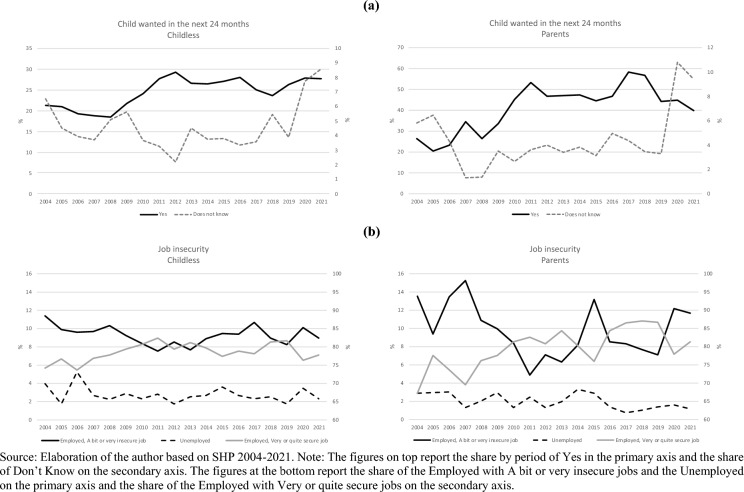
Intention to have a child during the next 24 months and job insecurity by wave and parity. *Source* Elaboration of the author based on SHP 2004–2021. Note: The figures on top report the share by period of Yes in the primary axis and the share of Don’t Know on the secondary axis. The figures at the bottom report the share of the Employed with A bit or very insecure jobs and the Unemployed on the primary axis and the share of the Employed with Very or quite secure jobs on the secondary axis

Figure 5 (panel a) illustrates the distribution of childbearing intentions over time by parity. Until 2012, childbearing intentions displayed a positive trend both among childless and parents, then first birth intentions stabilized and declined after 2016. Second birth intentions displayed a slightly more pronounced drop but only after 2017. Both among childless and parents, the share of respondents reporting an uncertain answer declined at the beginning of the observed period but then doubled in 2020 with respect to the period 2014–19. Figure 5 (panel b) illustrates the distribution of employment status and perceived job insecurity over time by parity. Employment conditions, both objective and subjective, deteriorated with some delay after the onset of the Great Recession. The proportion of unemployed, despite being rather small in absolute terms, increased after 2012 and remained higher than before especially among childless individuals, before peaking again in 2020. Among the employed, those who report having a quite or very insecure job increased after 2011 peaking first in 2015–2016 and then again in 2020 especially among parents.

**Fig. 6:**
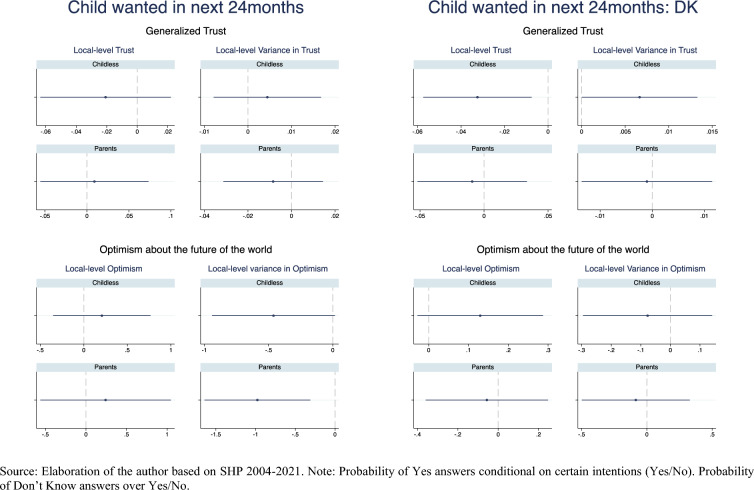
Fixed effect linear probability model of positive (Yes) and uncertain (Don’t Know) intentions. Average marginal effects of social climate and social uncertainty. *Source* Elaboration of the author based on SHP 2004–2021. *Note* Probability of Yes answers conditional on certain intentions (Yes/No). Probability of Don’t Know answers over Yes/No

The left panel of Fig. 6 reports the results of the Linear Probability Fixed Effects models (Model 1, Tables 3, 4, 5, 6, 7, 8, 9 and 10) of the association between social climate and social predictability measured through generalized trust and the probability of expressing positive or uncertain first or second child intentions. The probability of expressing positive intention to have a first child is not significantly influenced by the overall level of generalized trust and its unpredictability, irrespectively of the number and kind of controls included (Tables 3, 4). Yet, among childless respondents, an increasingly positive social climate reduces uncertain answers, while increasing uncertainty about the social climate increases uncertain answers (Tables 5, 6). No association is detected instead among parents. Controlling for individual-level employment uncertainty does not alter the estimates of social climate and social uncertainty in any model (Models 3 in Tables 3, 4, 5, 6). As found elsewhere, entering joblessness (especially unemployment) reduces first birth, but not second births, intention. Notably, it does not affect the probability of expressing uncertain intentions. Furthermore, there are no significant differences in childbearing intentions between employed respondents who move from a secure to a more insecure job.

On the right panel, Fig. 6 reports the association between social climate and social predictability measured through optimism about the future and first and second birth positive and uncertain short-time intentions (Model 1, Tables 7, 8, 9, 10). The probability of expressing positive intentions to have a first or second child is weakly positively associated to a more optimistic social climate (point estimates are not statistically significant for the first child but they are for the second). Much stronger and statistically significant is the negative effect of the unpredictability of the social climate on the respondents’ probability of wanting a first and especially a second child in the next 24 months (Tables 7, 8). Controlling for individual-level labor market insecurity does not alter these associations for the childless, and even make the point estimates stronger for parents (Models 3, Tables 7, 8, 9, 10). Net of income, positively associated to the intention to have a first child, employment uncertainty does not affect first childbearing intentions, while it is even positively associated to intending to have a second child^11^. In contrast, the probability of uncertain answers increases with a more optimistic social climate and decreases with the greater unpredictability of the social climate, although most point estimates do not reach statistical significance (Tables 9, 10). Yet, additional analyses (Table 11) suggest that the increase in D/K answers brought about by a more positive social climate, comes at the expenses of the negative and not of positive answers.

**Fig. 7:**
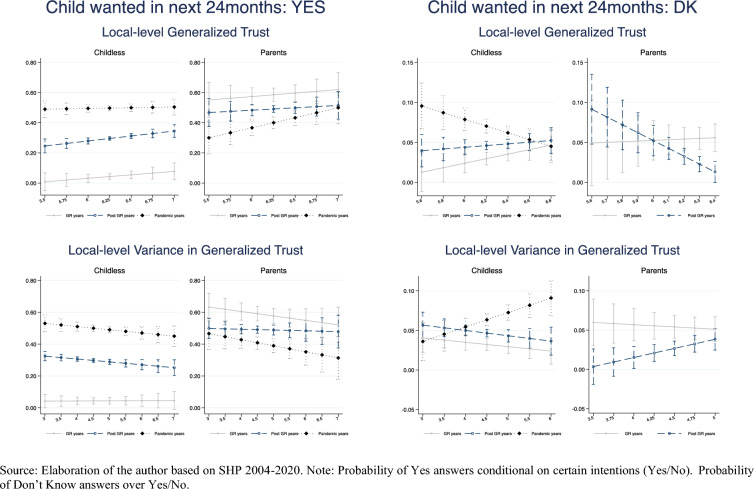
Fixed effect linear probability model of positive (Yes) and uncertain (Don’t Know) intentions. Predicted probability by period, social climate and social uncertainty. *Source* Elaboration of the author based on SHP 2004–2021. *Note* Probability of Yes answers conditional on certain intentions (Yes/No). Probability of Don’t Know answers over Yes/No

Figure 7 illustrates the predicted probability of expressing positive or uncertain first or second childbearing intentions depending on the canton mean and variance of generalized trust by period (see Table 12 for full models). Here, due to the very small number of observations I can only control for individual-level fixed effects, hence estimates have to be interpreted more cautiously. The probability of intending to have a child in the next 24 months seems to be particularly positively influenced by the social climate and its predictability among childless respondents during the post Great Recession years, while among parents during the years around the pandemic. On the contrary, the probability of expressing uncertain intentions seems more sensible to the worsening of the social climate and the increase in social uncertainty during the pandemic years for the childless and during the post Great Recession period for parents.

## Discussion

The study assessed the role of social climate and social uncertainty on childbearing decisions in contemporary low fertility societies. The argument tested is whether in times of endemic insecurity, beyond the more commonly investigated economic dimension of uncertainty—whose influence on both reproductive intentions and behavior has been extensively demonstrated (Alderotti et al., 2021)—the more social dimension of uncertainty also plays a role in hindering childbirths. The study builds on a conceptual framework that sees major crises as also social phenomena. Empirically, thanks to the very rich and timely longitudinal data available for Switzerland (SHP 2004–2021), I evaluate the influence of worsening social climate (lowered trust and optimism about the future in the canton of residence) and increasing social uncertainty (increased volatility of trust and optimism about the future in the canton of residence) on childless’ and parents’ short-term childbearing intentions. Furthermore, I test whether these associations differ not only across parities, but also across periods around two major recent crises: The Great Recession and the Covid-19 pandemic. Overall, results from the different models presented show either associations in the expected direction or non-significant associations. Net of sociodemographic, economic objective and subjective factors and unobserved (individual and local-level) determinants of childbearing decisions, a more positive and more predictable social climate increase—with varying degree depending on parity, indicator, and period—both the certainty and the positivity of short-time childbearing intentions.

In accordance with previous studies, the relationship between economic uncertainty and childbirth intentions varies across parity. I find that men and women exposed to greater individual-level economic uncertainty display more negative first, but not second, birth intentions. This not the case for social climate and social uncertainty. What varies mostly by parity is the type of reaction a changing social climate triggers in different historical periods. Childless respondents reacted to the more negative climate and its growing unpredictability during the post Great Recession period revising intentions from positive to negative. During the same period, parents instead revised their intentions towards more uncertain answers. The opposite happened during the pandemic: childless individuals expressed more uncertain intentions in response to the more negative and unpredictable social climate, while parents expressed more negative intentions. This is likely due to the difference between the two crises in terms of which group faced the greatest social adversities. Young childless individuals where more strongly and more permanently penalized by the Great Recession as much as parents were during the pandemic.

The study does not come without limitations. First, while short-term fertility intentions are seen as direct predecessors of reproductive behavior, economic, cultural and institutional constraints affect their realization (Billingsley & Ferrarini, 2014; Dommermuth et al., 2015; Hanappi et al., 2017; Ní Bhrolcháin & Beaujouan, 2019; Philipov et al., 2006). The estimated effects of social climate and uncertainties on actual reproductive behavior are, therefore, likely to be even greater than the ones on intentions identified here, as social uncertainties may act additionally as constraints on the realization of intentions. Moreover, even in western countries some (few) births are still unplanned, but the latter remain unaccounted in this study (Wellings et al., 2013). Second, the evaluation of the pandemic effects is based on few and early data points. It would be important, as new data become available, to verify the potential medium-to-long-term effects of the Covid-19 crisis on childbearing intentions. Third, due to the rapidly shrinking number of observations, heterogeneities by gender could not be investigated, despite a recent large body of research on the determinants of fertility intentions suggesting that they also depend on gender-related factors (Begall & Mills, 2011; Neyer et al., 2013). Finally, the availability of the variable Optimism about the future limited only to the years 2006 and 2012 represents a concern. More than two data points would have been useful to more accurately detect responses to change over time, but more importantly, these waves of data collection are way too early to detect any possible impact of the major crises episodes that characterized the past two decades. The results presented on optimism about the future are hence likely to represent lower bounds of the effects of social climate and social uncertainty on childbearing intentions.

Despite these limitations, results show that both social climate and social uncertainty, in the form of declining levels and increasing dispersion of generalized trust or optimism about the future in the local area of residence, correlate with the intention to have a child and with the degree of certainty of such intentions. The indicator reflecting the character of social interactions and morality (i.e., generalized trust) seems to influence more the choice between *certain versus uncertain* childbearing intentions. The indicator explicitly reflecting expectations about the future of the world instead more strongly influence the alternative between certain *positive versus negative* intentions. Overall it seems that, differently from economic uncertainty, both childless and one-child parents are affected by the quality of the social climate and its uncertainty (with the important parity-period interactions mentioned above). Finally, results tend to support the argument that the worsening social climate and increasing social uncertainty represent medium-to-long-term consequences of the Great Recession for reproductive decisions. The social features of the Covid-19 crisis instead appear as having made the social climate more consequential for the uncertainty of childbearing intentions. An important avenue for future research that could shed more light on the heterogeneities by indicator, parity and period identified in this study, would be to investigate the potential mechanisms mediating the association between social climate and social uncertainty and childbearing decisions, for instance the role of union formation or of outsourcing of care services.

All in all, the results of the study are likely to extend beyond the Swiss context and estimates likely represent a lower bound of the association between social climate and social uncertainty and childbearing intentions. Being Switzerland, in comparative terms, a country with relatively high levels of generalized trust and where the Great Recession hit relatively mildly, the long-term social effects of the financial crisis may be more limited than in other contexts, i.e., Southern European countries, for which, however, suitable data are not available. The study’s findings are likely to extend to most low fertility countries, as they have shared the major economic, social and political events of the past decades which, although with different degrees, have been affected by growing uncertainties since the onset of the Great Recession fifteen years ago. More broadly, the study highlights the importance of extending the conceptualization of uncertainty in contemporary societies to understand their importance in affecting past and future childbearing decisions.

See Tables 2, 3, 4, 5, 6, 7, 8, 9, 10, 11, 12.

**Table 2:** Tabulation of cantons *Source* Elaboration of the author based on SHP 2004–2021

	Trust models (waves 2004–2021)				Optimism Models (waves 2006, 2012)			
	Childless		Parents		Childless		Parents	
Canton	*N*	%	*N*	%	*N*	%	*N*	%
AG Argovia	1257	9.47	303	6.74	102	8.88	36	9.28
AI Appenzell Inner-Rhodes	14	0.11	11	0.24	3	0.26	1	0.26
AR Appenzell Outer-Rhodes	94	0.71	16	0.36	5	0.44	4	1.03
BE Berne	1676	12.63	552	12.28	151	13.14	38	9.79
BS Basle-Town	307	2.31	75	1.67	27	2.35	6	1.55
BL Basle-Country	408	3.07	163	3.63	35	3.05	12	3.09
FR Fribourg	567	4.27	236	5.25	55	4.79	25	6.44
GE Geneva	564	4.25	232	5.16	54	4.70	24	6.19
GL Glarus	70	0.53	15	0.33	7	0.61	2	0.52
GR Grisons	227	1.71	109	2.43	12	1.04	9	2.32
JU Jura	37	0.28	30	0.67			3	0.77
LU Lucerne	899	6.77	312	6.94	86	7.48	20	5.15
NE Neuchatel	559	4.21	185	4.12	60	5.22	11	2.84
NW Nidwalden	54	0.41	14	0.31	6	0.52	3	0.77
OW Obwalden	46	0.35	15	0.33	5	0.44		
SG St. Gall	622	4.69	173	3.85	49	4.26	17	4.38
SH Schaffhausen	83	0.63	37	0.82	4	0.35	3	0.77
SO Solothurn	431	3.25	131	2.91	35	3.05	13	3.35
SZ Schwyz	255	1.92	82	1.82	26	2.26	5	1.29
TG Thurgovia	283	2.13	90	2.00	23	2.00	5	1.29
TI Ticino	407	3.07	186	4.14	34	2.96	17	4.38
UR Uri	75	0.57	14	0.31	8	0.70		
VD Vaud	1438	10.83	471	10.48	112	9.75	41	10.57
VS Valais	355	2.67	166	3.69	38	3.31	12	3.09
ZG Zug	153	1.15	55	1.22	13	1.13	4	1.03
ZH Zurich	2391	18.02	821	18.27	199	17.32	77	19.85
Total	13,272	100.00	4494	100.00	1149	100.00	388	100.00

**Table 3:** Fixed effects models of Probability of wanting a child during the next 24 months (YES answer) by canton-level Generalized Trust. Childless *Source* Elaboration of the author based on SHP 2004–2021

First child						
	Mean			Variance		
	Model	Model	Model	Model	Model	Model
	(1)	(2)	(3)	(4)	(5)	(6)
Community generalized trust (mean-centered)	0.004	0.005	0.005			
	(−0.008–0.017)	(−0.00–0.018)	(−0.007–0.018)			
Community variance in generalized trust (mean-centered)				−0.021	0.006	0.004
				(−0.064–0.022)	(−0.037–0.049)	(−0.039–0.047)
Employment uncertainty						
(ref: Employed, very or quite secure)						
Employed, a bit or very insecure job			0.014			0.014
			(−0.010–0.038)			(-0.011–0.038)
Unemployed			−0.057***			-0.056***
			(−0.091– −0.022)			(-0.091– −0.021)
Out of the LF			−0.022			-0.021
			(−0.061–0.018)			(-0.061–0.018)
Student			−0.027**			-0.027**
			(−0.049– −0.005)			(-0.049– −0.005)
Disposable income (mean-centered)			0.004***			0.004***
			(0.001–0.006)			(0.001–0.006)
Education (ref: upper secondary)						
Primary or low secondary		0.009	0.006		0.009	0.006
		(−0.030–0.048)	(−0.033–0.045)		(−0.030–0.048)	(−0.033–0.045)
Tertiary		0.018	0.012		0.018	0.012
		(−0.009–0.046)	(−0.016–0.040)		(−0.009–0.045)	(−0.016–0.040)
Age (ref: 30–34)						
15–19	−0.541***	−0.286***	−0.285***	−0.541***	−0.282***	−0.282***
	(−0.578– −0.504)	(−0.332– −0.239)	(−0.332– -0.239)	(−0.579– −0.504)	(−0.329– −0.235)	(−0.330– −0.235)
20–24	−0.508***	−0.319***	−0.318***	−0.509***	−0.317***	−0.316***
	(−0.541– −0.476)	(−0.357– −0.282)	(−0.355– −0.280)	(−0.541– −0.476)	(−0.354– −0.279)	(−0.353– −0.278)
25–29	−0.335***	−0.244***	−0.242***	−0.335***	−0.242***	−0.241***
	(−0.362– −0.308)	(−0.272– −0.215)	(−0.270– −0.214)	(−0.363– −0.308)	(−0.271– −0.214)	(−0.269– −0.212)
35–39	0.239***	0.143***	0.141***	0.239***	0.142***	0.139***
	(0.188–0.290)	(0.093–0.194)	(0.090–0.191)	(0.188–0.290)	(0.091–0.193)	(0.088–0.190)
40–44	0.169***	−0.009	−0.014	0.170***	−0.012	−0.016
	(0.085–0.252)	(−0.091–0.074)	(−0.096–0.069)	(0.086–0.254)	(−0.095–0.071)	(−0.099–0.067)
Period (ref: 2004–2008)						
2009–2013		0.107***	0.102***		0.104***	0.099***
		(0.077–0.136)	(0.072–0.131)		(0.075–0.133)	(0.070–0.128)
2014–2018		0.241***	0.237***		0.238***	0.234***
		(0.201–0.282)	(0.196–0.277)		(0.198–0.278)	(0.194–0.273)
2019–2021		0.353***	0.347***		0.353***	0.347***
		(0.304–0.402)	(0.299–0.396)		(0.304–0.402)	(0.298–0.395)
Community FE	Yes	Yes	Yes	Yes	Yes	Yes
Constant	0.513***	0.234***	0.243***	0.516***	0.230***	0.240***
	(0.461–0.564)	(0.169–0.298)	(0.178–0.307)	(0.463–0.568)	(0.165–0.295)	(0.175–0.305)
Observations	12,631	12,631	12,631	12,631	12,631	12,631
R-squared	0.172	0.191	0.194	0.172	0.190	0.194
No. of idpers	4407	4407	4407	4407	4407	4407

Robust confidence intervals in parentheses, *** *p*<0.01, ** *p*<0.05, * *p*<0.1

**Table 4:** Fixed effects models of Probability of wanting a child during the next 24 months (YES answer) by canton-level Generalized Trust. Parents *Source* Elaboration of the author based on SHP 2004–2021

Second child						
	Mean			Variance		
	Model	Model	Model	Model	Model	Model
	(1)	(2)	(3)	(4)	(5)	(6)
Community Generalized Trust (mean-centered)	−0.001	−0.013	−0.012			
	(−0.024–0.022)	(−0.037–0.011)	(−0.036–0.011)			
Community Variance in Generalized Trust (mean-centered)				0.009	0.032	0.039
				(−0.056–0.073)	(−0.031–0.095)	(−0.024–0.101)
Employment Uncertainty						
(ref: Employed, Very or quite secure)						
Employed, A bit or very insecure job			0.013			0.014
			(−0.026–0.053)			(−0.026–0.053)
Unemployed			−0.023			−0.022
			(−0.115–0.069)			(−0.114–0.070)
Out of the LF			−0.013			−0.012
			(−0.076–0.050)			(−0.075–0.051)
Student			0.103			0.102
			(−0.115–0.321)			(−0.116–0.319)
Disposable Income (mean-centered)			−0.011***			−0.011***
			(−0.015– −0.006)			(−0.015– −0.006)
Education (ref: Upper Secondary)						
Primary or Low Secondary		−0.361***	−0.362***		-0.360***	−0.361***
		(-0.571– −0.151)	(−0.579– −0.145)		(-0.569– −0.151)	(−0.577– −0.145)
Tertiary		0.062	0.068		0.061	0.068
		(-0.072–0.196)	(−0.066–0.203)		(−0.073–0.196)	(−0.067–0.202)
Age (ref: 30–34)						
15–19	−0.117	−0.070	−0.070	−0.117	−0.072	−0.071
	(−0.485–0.251)	(−0.445–0.305)	(−0.418–0.277)	(−0.484–0.251)	(−0.445–0.302)	(−0.417–0.276)
20–24	−0.401***	−0.420***	−0.416***	−0.400***	−0.419***	−0.414***
	(−0.558–−0.245)	(−0.584–−0.256)	(−0.581–−0.252)	(−0.556–−0.244)	(−0.582–−0.256)	(−0.578–−0.250)
25–29	−0.071	−0.113**	−0.108*	−0.070	−0.114**	−0.108*
	(−0.165–0.024)	(−0.207–−0.019)	(−0.201–−0.014)	(−0.165–0.025)	(−0.209–−0.020)	(−0.202–−0.015)
35–39	−0.092**	−0.008	−0.008	−0.092**	−0.008	−0.009
	(−0.153–−0.030)	(−0.068–0.053)	(−0.070–0.053)	(−0.154–−0.031)	(−0.068–0.053)	(−0.070–0.052)
40–44	−0.269***	−0.082*	−0.082*	−0.270***	−0.083*	−0.083*
	(−0.344–−0.195)	(−0.159–−0.005)	(−0.159–−0.005)	(−0.345–−0.195)	(−0.160–−0.006)	(−0.160–−0.006)
Period (ref: 2004–2008)						
		−0.067**	−0.064*		−0.062*	−0.059*
2009–2013		(−0.122–−0.011)	(−0.119–−0.008)		(−0.117–−0.007)	(−0.114–−0.004)
		−0.095*	−0.096*		−0.088*	−0.089*
2014–2018		(−0.179–−0.012)	(−0.180–−0.013)		(−0.171–−0.006)	(−0.172–−0.007)
		−0.123**	−0.124**		−0.118*	−0.118*
2019–2021		(−0.225–−0.021)	(−0.227–−0.022)		(−0.221–−0.016)	(−0.221–−0.016)
Age of first child (mean−centered)		−0.020***	−0.018***		−0.020***	−0.018***
		(−0.028–−0.011)	(−0.027–−0.009)		(−0.028–−0.011)	(−0.027–−0.010)
Community FE	Yes	Yes	Yes	Yes	Yes	Yes
Constant	−0.132	−0.118	−0.036	−0.134	−0.125	−0.045
	(−0.328–0.064)	(−0.329–0.094)	(−0.255–0.182)	(−0.332–0.063)	(−0.337–0.087)	(−0.264–0.174)
Observations	4274	4274	4274	4274	4274	4274
R−squared	0.069	0.084	0.090	0.070	0.084	0.090
Number of idpers	1688	1688	1688	1688	1688	1688

**Table 5:** Fixed effects models of Probability of being uncertain about wanting a child during the next 24 months (D/K answer) by canton−level Generalized Trust Childless *Source* Elaboration of the author based on SHP 2004–2021

First child						
	Mean			Variance		
	Model	Model	Model	Model	Model	Model
	(1)	(2)	(3)	(4)	(5)	(6)
Community generalized trust (mean−centered)	−0.032**	−0.030*	−0.030*			
	(−0.057–−0.008)	(−0.055–−0.004)	(−0.055–−0.004)			
Community variance in generalized trust (mean−centered)				0.007*	0.005	0.005
				(0.000–0.013)	(−0.002–0.012)	(−0.002–0.012)
Employment uncertainty						
(ref: Employed, very or quite secure)						
Employed, a bit or very insecure job			0.001			0.001
			(−0.014–0.017)			(−0.015–0.016)
Unemployed			−0.000			−0.000
			(−0.023–0.023)			(−0.024–0.023)
Out of the LF			0.017			0.017
			(−0.008–0.043)			(−0.008–0.043)
Student			−0.008			−0.008
			(−0.020–0.004)			(−0.020–0.004)
Disposable income (mean−centered)			0.000			0.000
			(−0.001–0.001)			(−0.001–0.001)
Education (ref: upper secondary)						
Primary or low secondary		0.003	0.003		0.003	0.003
		(−0.013–0.019)	(−0.012–0.019)		(−0.013–0.019)	(−0.013–0.018)
Tertiary		0.010	0.009		0.010	0.009
		(−0.004–0.024)	(−0.006–0.023)		(−0.004–0.024)	(−0.006–0.023)
Age (ref: 30–34)						
15–19	−0.075***	−0.072***	−0.071***	−0.074***	−0.069***	−0.068***
	(−0.091–−0.058)	(−0.097–−0.048)	(−0.096–−0.047)	(−0.090–−0.057)	(−0.093–−0.045)	(−0.092–−0.044)
20–24	−0.066***	−0.063***	−0.062***	−0.065***	−0.060***	−0.060***
	(−0.081–−0.051)	(−0.083–−0.042)	(−0.083–−0.041)	(−0.080–−0.050)	(−0.080–−0.040)	(−0.080–−0.039)
25–29	−0.037***	−0.036***	−0.036***	−0.036***	−0.035***	−0.035***
	(−0.050–−0.024)	(−0.051–−0.021)	(−0.051–−0.021)	(−0.049–−0.023)	(−0.050–−0.020)	(−0.050–−0.020)
35–39	0.001	0.001	0.001	0.000	−0.000	−0.000
	(−0.023–0.026)	(−0.024–0.026)	(−0.023–0.026)	(−0.024–0.025)	(−0.025–0.025)	(−0.025–0.025)
40–44	−0.050*	−0.049*	−0.048*	−0.052**	−0.053**	−0.053**
	(−0.092–−0.007)	(−0.092–−0.005)	(−0.092–−0.005)	(−0.095–−0.010)	(−0.096–−0.010)	(−0.096–−0.009)
Period (ref: 2004–2008)						
2009–2013		−0.006	−0.007		−0.006	−0.006
		(−0.022–0.009)	(−0.022–0.009)		(−0.021–0.010)	(−0.022–0.010)
2014–2018		−0.006	−0.007		−0.004	−0.004
		(−0.027–0.015)	(−0.028–0.014)		(−0.025–0.017)	(−0.026–0.017)
2019–2021		−0.002	−0.003		0.000	−0.000
		(−0.029–0.025)	(−0.030–0.024)		(−0.027–0.027)	(−0.027–0.027)
Community FE	Yes	Yes	Yes	Yes	Yes	Yes
Constant	0.098***	0.095***	0.096***	0.093***	0.088***	0.089***
	(0.071–0.126)	(0.059–0.131)	(0.060–0.132)	(0.067–0.120)	(0.053–0.123)	(0.054–0.124)
Observations	13,272	13,272	13,272	13,272	13,272	13,272
R−squared	0.013	0.013	0.013	0.012	0.013	0.013
Number of idpers	4547	4547	4547	4547	4547	4547

**Table 6:** Fixed effects models of Probability of being uncertain about wanting a child during the next 24 months (D/K answer) by canton−level Generalized Trust Parents *Source* Elaboration of the author based on SHP 2004–2021

Second child						
	Mean			Variance		
	Model	Model	Model	Model	Model	Model
	(1)	(2)	(3)	(4)	(5)	(6)
Community generalized trust (mean−centered)	−0.009	−0.003	−0.004			
	(−0.052–0.034)	(−0.046–0.039)	(−0.046–0.039)			
Community variance in generalized trust (mean−centered)				0.001	0.002	0.002
				(−0.011–0.014)	(−0.012–0.015)	(−0.011–0.016)
Employment uncertainty (ref: employed, very or quite secure)						
Employed, a bit or very insecure job			0.000			0.000
			(−0.022–0.022)			(−0.022–0.023)
Unemployed			−0.022			−0.022
			(−0.065–0.021)			(−0.065–0.021)
Out of the LF			0.038			0.038
			(−0.006–0.083)			(−0.006–0.083)
Student			0.092*			0.091*
			(0.003–0.181)			(0.003–0.180)
Disposable income (mean−centered)			−0.003			−0.003
			(−0.006–0.000)			(−0.006–0.000)
Education (ref: upper secondary)						
Primary or low secondary		−0.068	−0.073		−0.068	−0.072
		(−0.225–0.088)	(−0.226–0.081)		(−0.224–0.088)	(−0.226–0.081)
Tertiary		0.017	0.018		0.017	0.018
		(−0.066–0.100)	(−0.064–0.101)		(−0.066–0.100)	(−0.064–0.101)
Age (ref: 30–34)						
15–19	0.023	0.039	0.026	0.024	0.039	0.026
	(−0.081–0.127)	(−0.059–0.138)	(−0.085–0.138)	(−0.082–0.129)	(−0.060–0.138)	(−0.087–0.138)
20–24	0.071	0.069	0.064	0.072	0.069	0.064
	(−0.013–0.155)	(−0.012–0.149)	(−0.018–0.147)	(−0.012–0.157)	(−0.012–0.150)	(−0.019–0.147)
25–29	0.055**	0.044*	0.043*	0.055**	0.044*	0.043*
	(0.015–0.094)	(0.005–0.083)	(0.004–0.082)	(0.015–0.094)	(0.004–0.083)	(0.003–0.082)
35–39	−0.007	0.015	0.015	−0.007	0.015	0.015
	(−0.027–0.014)	(−0.008–0.039)	(−0.008–0.039)	(−0.027–0.014)	(−0.008–0.039)	(−0.008–0.039)
40–44	−0.059***	−0.013	−0.013	−0.060***	−0.013	−0.013
	(−0.084–−0.034)	(−0.048–0.023)	(−0.049–0.022)	(−0.086–−0.034)	(−0.048–0.023)	(−0.049–0.022)
Period (ref: 2004–2008)						
2009–2013		0.010	0.012		0.010	0.013
		(−0.012–0.031)	(−0.010–0.033)		(−0.012–0.032)	(−0.010–0.035)
2014–2018		0.025	0.026		0.026	0.028
		(−0.011–0.060)	(−0.009–0.062)		(−0.011–0.062)	(−0.008–0.064)
2019–2021		0.015	0.018		0.016	0.018
		(−0.034–0.064)	(−0.031–0.067)		(−0.034–0.065)	(−0.031–0.067)
Age of first child (mean−centered)		−0.008***	−0.007***		−0.008***	−0.007***
		(−0.012–−0.004)	(−0.012–−0.003)		(−0.012–−0.004)	(−0.012–−0.003)
Community FE	Yes	Yes	Yes	Yes	Yes	Yes
Constant	−0.036	−0.019	−0.017	−0.037	−0.019	−0.036
	(−0.167–0.095)	(−0.149–0.111)	(−0.140–0.107)	(−0.167–0.093)	(−0.149–0.110)	(−0.167–0.095)
Observations	4,494	4,494	4,494	4,494	4,494	4,494
R−squared	0.021	0.025	0.017	0.021	0.025	0.021
No. of idpers	1752	1752	1752	1752	1752	1752

**Table 7:** Fixed effects models of Probability of wanting a child during the next 24 months (YES answer) by local Optimism about the future. Childless *Source* Elaboration of the author based on SHP 2004–2021

First child						
	Mean			Variance		
	Model	Model	Model	Model	Model	Model
	(1)	(2)	(3)	(4)	(5)	(6)
Community optimism about the future (mean−centered)	0.207	0.115	0.028			
	(−0.352–0.767)	(−0.455–0.685)	(−0.502–0.558)			
Community variance in optimism about the future (mean−centered)				−0.462	−0.682**	−0.615**
				(−0.941–0.017)	(−1.119–−0.246)	(−1.052–−0.179)
Employment uncertainty						
(ref: Employed, very or quite secure)						
Employed, a bit or very insecure job			0.180			0.188
			(−0.055–0.416)			(−0.017–0.393)
Unemployed			0.012			0.037
			(−0.269–0.292)			(−0.205–0.278)
Out of the LF			−0.015			0.022
			(−0.260–0.229)			(−0.234–0.278)
Student			−0.014			−0.011
			(−0.213–0.185)			(−0.205–0.184)
Disposable income (mean−centered)			0.017*			0.012
			(0.001–0.032)			(−0.003–0.027)
Education (ref: upper secondary)						
Primary or low secondary		−0.604***	−0.563***		−0.556***	−0.534***
		(−0.801–−0.406)	(−0.774–−0.353)		(−0.775–−0.338)	(−0.764–−0.304)
Tertiary		−0.214*	−0.192		−0.154	−0.147
		(−0.420–−0.008)	(−0.398–0.015)		(−0.360–0.052)	(−0.351–0.057)
Age (ref: 30–34)						
15–19	−0.690***	0.039	−0.024	−0.866***	0.209	0.140
	(−1.001–−0.378)	(−0.574–0.653)	(−0.632–0.585)	(−1.166–−0.565)	(−0.398–0.817)	(−0.475–0.755)
20–24	−0.793***	−0.433	−0.453*	−0.925***	−0.303	−0.333
	(−1.033–−0.553)	(−0.896–0.030)	(−0.906–−0.001)	(−1.153–−0.698)	(−0.753–0.147)	(−0.784–0.119)
25–29	−0.461***	−0.288*	−0.283*	−0.530***	−0.244*	−0.247*
	(−0.620–−0.302)	(−0.543–−0.034)	(−0.533–−0.034)	(−0.680–−0.379)	(−0.484–−0.004)	(−0.488–−0.006)
35–39	−0.042	−0.246	−0.234	0.015	−0.309*	−0.303*
	(−0.306–0.222)	(−0.552–0.060)	(−0.534–0.065)	(−0.226–0.257)	(−0.599–−0.019)	(−0.595–−0.011)
40–44	−0.365*	−0.839**	−0.838***	−0.191	−0.899***	−0.903***
	(−0.689–−0.041)	(−1.385–−0.293)	(−1.365–−0.310)	(−0.523–0.141)	(−1.428–−0.371)	(−1.422–−0.383)
Wave 2012 (ref: 2006)		0.302	0.265		0.465***	0.414**
		(−0.004–0.608)	(−0.032–0.562)		(0.171–0.760)	(0.113–0.715)
Community FE	Yes	Yes	Yes	Yes	Yes	Yes
Constant	0.757***	0.629**	0.600**	0.783***	0.389	0.389
	(0.554–0.960)	(0.211–1.048)	(0.187–1.014)	(0.596–0.970)	(−0.034–0.811)	(−0.039–0.818)
Observations	1,116	1,116	1,116	1,116	1,116	1,116
R−squared	0.470	0.535	0.551	0.481	0.558	0.569
No. of idpers	999	999	999	999	999	999

**Table 8:** Fixed effects models of Probability of wanting a child during the next 24 months (YES answer) by local Optimism about the future Parents *Source* Elaboration of the author based on SHP 2004–2021

Second child						
	Mean			Variance		
	Model	Model	Model	Model	Model	Model
	(1)	(2)	(3)	(4)	(5)	(6)
Community optimism about the future (mean−centered)	0.242	0.678	1.260***			
	(−0.563–1.046)	(−0.091–1.448)	(0.737–1.784)			
Community variance in optimism about the future (mean−centered)				−1.208***	−0.924**	−1.058***
				(−1.930–−0.486)	(−1.623–−0.225)	(−1.576–−0.540)
Employment uncertainty						
(ref: Employed, very or quite secure)						
Employed, a bit or very insecure job			0.663***			0.464***
			(0.514–0.813)			(0.324–0.604)
Unemployed						
Out of the LF			0.800***			0.771***
			(0.650–0.949)			(0.591–0.951)
Student			−1.312***			−0.874*
			(−2.030–−0.594)			(−1.691–−0.058)
Disposable income (mean−centered)			0.003			0.010
			(−0.024–0.029)			(−0.020–0.040)
Education (ref: upper secondary)						
Primary or low secondary						
Tertiary		−0.022	0.161		−0.017	0.056
		(−0.722–0.678)	(−0.526–0.848)		(−0.812–0.778)	(−0.704–0.816)
Age (ref: 30–34)						
15–19						
20–24						
25–29	−0.504	−9.720***	−0.565***	−0.821**	−8.098**	−0.840***
	(−1.152–0.143)	(−14.320–−5.120)	(−0.872–−0.259)	(−1.455–−0.187)	(−13.562–−2.633)	(−1.211–−0.470)
35–39	−0.257	0.347**	0.477***	−0.101	0.313**	0.366***
	(−0.527–0.012)	(0.088–0.606)	(0.285–0.669)	(−0.333–0.131)	(0.069–0.558)	(0.154–0.578)
40–44	−0.489***	0.752***	0.704***	−0.152	0.733***	0.621***
	(−0.781–−0.197)	(0.386–1.117)	(0.383–1.025)	(−0.342–0.037)	(0.332–1.134)	(0.230–1.012)
Wave 2012 (ref: 2006)		−3.397***	−0.772***		−2.690***	−0.498***
		(−4.730–−2.064)	(−1.050–−0.494)		(−4.263–−1.118)	(−0.765–−0.231)
Age of first child (mean−centered)		0.430***			0.345**	
		(0.208–0.651)			(0.081–0.608)	
Community FE	Yes	Yes	Yes	Yes	Yes	Yes
Constant	0.619***	0.584**	0.226	0.427***	0.461	0.207
	(0.418–0.821)	(0.167–1.000)	(−0.144–0.597)	(0.280–0.574)	(−0.031–0.953)	(−0.206–0.620)
Observations	372	372	372	372	372	372
R−squared	0.476	0.646	0.793	0.569	0.673	0.789
No. of idpers	337	337	337	337	337	337

**Table 9:** Fixed effects models of Probability of being uncertain about wanting a child during the next 24 months (D/K answer) by local Optimism about the future Childless *Source* Elaboration of the author based on SHP 2004–2021

First child						
	Mean			Variance		
	Model	Model	Model	Model	Model	Model
	(1)	(2)	(3)	(4)	(5)	(6)
Community optimism about the future (mean−centered)	0.130	0.207*	0.235*			
	(−0.028–0.287)	(0.004–0.411)	(0.036–0.433)			
Community variance in optimism about the future (mean−centered)				−0.077	−0.031	−0.053
				(−0.295–0.141)	(−0.279–0.216)	(−0.301–0.195)
Employment uncertainty						
(ref: Employed, very or quite secure)						
Employed, a bit or very insecure job			0.134			0.135
			(−0.001–0.270)			(−0.002–0.272)
Unemployed			0.004			0.011
			(−0.073–0.080)			(−0.060–0.082)
Out of the LF			0.025			0.026
			(−0.037–0.087)			(−0.050–0.101)
Student			−0.025			−0.017
			(−0.072–0.022)			(−0.059–0.025)
Disposable income ( Thousands mean−centered)			−0.003			−0.002
			(−0.014–0.008)			(−0.013–0.008)
Education (ref: upper secondary)						
Primary or low secondary		−0.015	−0.033		−0.003	−0.015
		(−0.048–0.018)	(−0.084–0.017)		(−0.034–0.027)	(−0.060–0.029)
Tertiary		0.060*	0.046		0.067*	0.058
		(0.006–0.114)	(−0.012–0.105)		(0.008–0.126)	(−0.001–0.117)
Age (ref: 30–34)						
15–19	0.061	−0.234*	−0.224*	0.003	−0.226*	−0.213
	(−0.029–0.150)	(−0.445–−0.022)	(−0.447–−0.000)	(−0.093–0.100)	(−0.437–−0.015)	(−0.444–0.019)
20–24	0.050	−0.159*	−0.155*	0.007	−0.152*	−0.143
	(−0.026–0.126)	(−0.308–−0.010)	(−0.307–−0.003)	(−0.074–0.088)	(−0.298–−0.006)	(−0.296–0.011)
25–29	0.037	−0.076	−0.088*	0.016	−0.075*	−0.082*
	(−0.044–0.118)	(−0.155–0.003)	(−0.172–−0.003)	(−0.068–0.100)	(−0.148–−0.002)	(−0.161–−0.003)
35–39	−0.136	−0.025	−0.023	−0.107	−0.011	−0.011
	(−0.283–0.011)	(−0.146–0.096)	(−0.142–0.096)	(−0.236–0.022)	(−0.143–0.120)	(−0.144–0.121)
40–44	−0.194*	0.075	0.065	−0.141*	0.070	0.055
	(−0.378–−0.010)	(−0.127–0.277)	(−0.137–0.267)	(−0.263–−0.019)	(−0.138–0.278)	(−0.158–0.267)
Wave 2012 (ref: 2006)		−0.172*	−0.172*		−0.136*	−0.127
		(−0.318–−0.026)	(−0.317–−0.026)		(−0.271–−0.001)	(−0.277–0.022)
Community FE	Yes	Yes	Yes	Yes	Yes	Yes
Constant	0.049	0.184*	0.183**	0.062	0.156*	0.141
	(−0.017–0.114)	(0.024–0.343)	(0.030–0.336)	(−0.003–0.126)	(0.005–0.306)	(−0.012–0.293)
Observations	1,149	1,149	1,149	1,149	1,149	1,149
R−squared	0.113	0.145	0.175	0.111	0.134	0.162
No. of idpers	1024	1024	1024	1024	1024	1024

**Table 10:** Fixed effects models of Probability of being uncertain about wanting a child during the next 24 months (D/K answer) by local Optimism about the future Parents *Source* Elaboration of the author based on SHP 2004–2021

Second child						
	Mean			Variance		
	Model	Model	Model	Model	Model	Model
	(1)	(2)	(3)	(4)	(5)	(6)
Community optimism about the future (mean−centered)	−0.056	0.117	0.249			
	(−0.360–0.247)	(−0.332–0.566)	(−0.259–0.757)			
Community variance in optimism about the future (mean−centered)				−0.180	−0.163	−0.130
				(−0.578–0.217)	(−0.611–0.284)	(−0.559–0.299)
Employment uncertainty						
(ref: Employed, very or quite secure)						
Employed, a bit or very insecure job			0.134			0.093
			(−0.130–0.399)			(−0.149–0.335)
Unemployed						
Out of the LF			0.193			0.183
			(−0.126–0.512)			(−0.130–0.496)
Student			−0.027			0.037
			(−0.301–0.246)			(−0.271–0.345)
Disposable income ( Thousands mean−centered)			−0.020			−0.020
			(−0.044–0.004)			(−0.045–0.005)
Education (ref: upper secondary)						
Primary or low secondary						
Tertiary		0.274**	0.251*		0.275**	0.218*
		(0.053–0.495)	(0.018–0.484)		(0.065–0.484)	(0.009–0.426)
Age (ref: 30–34)						
15–19						
20–24						
25–29	0.001	1.358**	−0.052	−0.048	1.634**	−0.093
	(−0.013–0.015)	(0.253–2.462)	(−0.248–0.144)	(−0.153–0.058)	(0.391–2.878)	(−0.326–0.141)
35–39	0.062*	0.261**	0.267**	0.086	0.254**	0.244**
	(0.001–0.122)	(0.066–0.455)	(0.055–0.478)	(−0.004–0.177)	(0.069–0.439)	(0.057–0.431)
40–44	−0.123*	0.258**	0.297*	−0.077	0.254**	0.285*
	(−0.227–−0.019)	(0.067–0.449)	(0.027–0.566)	(−0.201–0.048)	(0.066–0.442)	(0.028–0.542)
Wave 2012 (ref: 2006)		0.196	−0.240*		0.318*	−0.201*
		(−0.048–0.440)	(−0.443–−0.037)		(0.021–0.616)	(−0.377–−0.026)
Age of first child (mean−centered)		−0.076**			−0.091**	
		(−0.137–−0.015)			(−0.159–−0.022)	
Community FE	Yes	Yes	Yes	Yes	Yes	Yes
Constant	0.095***	−0.122	−0.140	0.063	−0.148	−0.128
	(0.040–0.149)	(−0.251–0.007)	(−0.309–0.028)	(−0.010–0.136)	(−0.320–0.024)	(−0.303–0.048)
Observations	388	388	388	388	388	388
R−squared	0.190	0.252	0.362	0.197	0.256	0.357
No. of idpers	349	349	349	349	349	349

**Table 11:** FE models of Probability of DK with respect to YES and NO answers by local optimism *Source* Elaboration of the author based on SHP 2004–2021

	Model	Model
	(1)	(2)
	Pr(DK) vs Pr(No)	Pr(DK) vs Pr(Yes)
Community Optimism about the future (mean−centered)	−1.147*	0.165
	(−2.226–−0.069)	(−0.059–0.390)
Constant	0.107***	0.040***
	(0.102–0.112)	(0.038–0.041)
Observations	323	859
R−squared	0.143	0.022
No. of idpers	308	801

**Table 12:** Fixed effects linear probability models with period interaction *Source* Elaboration of the author based on SHP 2004–2021

	Pr(Yes)				Pr(DK)			
	Model	Model	Model	Model	Model	Model	Model	Model
	(1)	(2)	(3)	(4)	(1)	(2)	(3)	(4)
	Childless	Parents	Childless	Parents	Childless	Parents	Childless	Parents
Community Generalized Trust (mean−centered)	0.073**	−0.020			−0.048**	−0.046		
	(0.014–0.133)	(−0.142–0.102)			(−0.085–−0.010)	(−0.106–0.014)		
Period								
2004–2008	−0.420***	0.252***	−0.418***	0.251***	−0.045***	0.095***	−0.039***	0.093***
	(−0.460–−0.379)	(0.165–0.339)	(−0.461–−0.376)	(0.162–0.340)	(−0.065–−0.025)	(0.062–0.127)	(−0.060–−0.018)	(0.059–0.126)
2009–2013	−0.236***	0.102***	−0.233***	0.101***	−0.041***	0.068***	−0.040***	0.066***
	(−0.263–−0.209)	(0.041–0.163)	(−0.260–−0.206)	(0.040–0.163)	(−0.056–−0.027)	(0.039–0.098)	(−0.055–−0.026)	(0.036–0.096)
2014–2018	REF	REF	REF	REF	−0.024***	0.046***	−0.024***	0.046***
					(−0.035–−0.013)	(0.022–0.069)	(−0.036–−0.013)	(0.023–0.070)
2019–2021	0.200***	−0.095***	0.199***	−0.093***	REF	REF	REF	REF
	(0.175–0.225)	(−0.144–−0.045)	(0.173–0.225)	(−0.145–−0.041)				
2004–2008*Community Generalized Trust	0.018	−0.043			0.043	0.020		
	(−0.069–0.105)	(−0.198–0.111)			(−0.003–0.089)	(−0.053–0.092)		
2009–2013*Community Generalized Trust	−0.018	0.006			0.064***	0.031		
	(−0.089–0.052)	(−0.128–0.141)			(0.025–0.102)	(−0.034–0.097)		
2019–2021*Community Generalized Trust	−0.053	0.111			0.048**	−0.035		
	(−0.114–0.009)	(−0.016–0.238)			(0.017–0.079)	(−0.085–0.015)		
Community Variance in Generalized Trust (mean−centered)			−0.019	0.006			0.018**	−0.005
			(−0.038–0.001)	(−0.035–0.048)			(0.003–0.033)	(−0.030–0.020)
2004–2008*Community Variance in Generalized Trust			0.001	0.009			−0.026**	0.009
			(−0.032–0.035)	(−0.044–0.063)			(−0.044–−0.009)	(−0.020–0.038)
2009–2013*Community Variance in Generalized Trust			0.017	−0.012			−0.021**	0.010
			(−0.010–0.044)	(−0.066–0.043)			(−0.037–−0.005)	(−0.019–0.040)
2014–2018*Community Variance in Generalized Trust							−0.023***	0.023
							(−0.037–−0.009)	(−0.000–0.046)
2019–2021*Community Variance in Generalized Trust			−0.003	−0.035				
			(−0.031–0.025)	(−0.094–0.025)				
Constant	0.355***	0.399***	0.354***	0.399***	0.075***	−0.001	0.074***	0.001
	(0.340–0.371)	(0.368–0.429)	(0.337–0.370)	(0.367–0.432)	(0.065–0.085)	(−0.020–0.018)	(0.065–0.083)	(−0.018–0.020)
Observations	12,631	4,274	12,631	4,274	13,272	4,494	13,272	4,494
R−squared	0.140	0.034	0.139	0.033	0.004	0.009	0.004	0.008
No. of idpers	4407	1688	4407	1688	4547	1752	4547	1752

Robust confidence intervals in parentheses, *** *p*<0.01, ** *p*<0.05, * *p*<0.1

## Footnotes

1 In the literature, short-term childbearing intentions are considered good proxies of fertility behavior (Morgan & Rackin 2010).

2 “Features of social organization such as networks, norms and trust that facilitate co-ordination and co-operation for mutual benefit” (Putnam, 1993, p. 167).

3 The WUI is based on the frequency of the world “uncertainty” in the quarterly Economists Intelligence Unit country reports. The Index appears highly synchronized across advanced economies (Ahir et al., 2022).

4 While the introduction of paid maternity leaves in 2005 (14 weeks’ maternity leave at 80% of prior earnings and two additional weeks of unpaid leave) was a significant improvement in family policies, Switzerland’s leave scheme remains minimal (Bertozzi & Gilardi, 2008; Valarino & Bernardi, 2010). The recently approved two-weeks paternity leave only helps men and women a little, and there is no paid parental leave for both partners.

5 Women still significantly reduce their participation on the labor market during the transition to parenthood and often do not return to full-time work afterwards (Widmer & Ritschard, 2009).

6 The 2020 wave of the SHP was fielded between September 2020 and February 2021, therefore several months after the start of the pandemic, during the second wave of infection spread. The 2021 wave was fielded between September 2021 and February 2022, therefore after the vaccination campaign and around the time when all the restrictions were lifted in Switzerland.

7 From 2015, the childbearing intention question is asked only to respondents who declare they want to have children in their life.

8 Here, following previous studies (Sandberg, 2006), I am making simplifying assumptions about the cognitive integration of information from the collectivity to the individual’s perception to construct a measure of social climate. See Sandberg, (2006) for a detailed illustration of assumptions and caveat of such aggregation method.

9 Although theoretically relevant, the distinction between full-time and part-time work did not yield any substantial additional information so for the sake of conciseness the distinction is disregarded.

10 The periodization is based on the trend in aggregate uncertainty (WUI) presented in Fig. 3 and GDP growth rates in Switzerland: the largest drop in GDP was registered in 2009 but GDP annual growth remained substantially below the pre-crisis average for a few years due to the Sovereign debt crisis that hit European countries in 2011–12. After 2014 Swiss’s GDP growth stabilized although it never returned to pre-2009 rates before dropping in 2020 even lower than in 2009 (World Bank data 2022).

11 The association with unemployment could not be estimated as there are not enough unemployed in the models for parents in waves 2006 and 2012.
